# A Three-Dimensional Finite Element Study on the Biomechanical Simulation of Various Structured Dental Implants and Their Surrounding Bone Tissues

**DOI:** 10.1155/2016/4867402

**Published:** 2016-01-19

**Authors:** Gong Zhang, Hai Yuan, Xianshuai Chen, Weijun Wang, Jianyu Chen, Jimin Liang, Peng Zhang

**Affiliations:** ^1^Guangzhou Institute of Advanced Technology, Chinese Academy of Sciences, Guangzhou 511458, China; ^2^Hospital of Stomatology, Sun Yat-sen University Hospital, Guangzhou 510000, China; ^3^Foshan Stomatology Hospital, Foshan 528000, China

## Abstract

*Background/Purpose*. This three-dimensional finite element study observed the stress distribution characteristics of 12 types of dental implants and their surrounding bone tissues with various structured abutments, implant threads, and healing methods under different amounts of concentrated loading.* Materials and Methods*. A three-dimensional geometrical model of a dental implant and its surrounding bone tissue was created; the model simulated a screw applied with a preload of 200 N or a torque of 0.2 N·m and a prosthetic crown applied with a vertical or an inclined force of 100 N. The Von Mises stress was evaluated on the 12 types of dental implants and their surrounding bone tissues.* Results*. Under the same loading force, the stress influence on the implant threads was not significant; however, the stress influence on the cancellous bone was obvious. The stress applied to the abutment, cortical bone, and cancellous bone by the inclined force applied to the crown was larger than the stress applied by the vertical force to the crown, and the abutment stress of the nonsubmerged healing implant system was higher than that of the submerged healing implant system.* Conclusion*. A dental implant system characterised by a straight abutment, rectangle tooth, and nonsubmerged healing may provide minimum value for the implant-bone interface.

## 1. Introduction

Since osseointegrated dental implants are introduced for the rehabilitation of the edentulous patient in the late 1960s, a tremendous awareness and subsequent demand have been arising in the field [1–3]. Recently, dental implants have been increasingly applied in oral rehabilitation and orthopedics used as replacements after the natural teeth are lost or partially damaged, which could restore human mastication functions [4]. Previous studies showed that dental implantation could have a high success rate: retention rate is in excess of 95% over a 5-year period if dental implants were correctly designed, manufactured, and inserted [5–7].

However, dental implant treatments are still failing frequently. One of the major causes of failure is that an artificial implant may never function as perfectly as the living tissues it replaces.

As a matter of fact, the success of dental implant is strongly affected by a number of biomechanical factors, including the type of loading, material properties of implant and prosthesis, implant geometry, surface structure, quality and quantity of surrounding bone, nature of implant-bone interface, and surgical procedures [8]. As far as implant shape is concerned, main design parameters affecting load transfer mechanisms include implant diameter and length of implant-bone interface [9], as well as thread pitch, shape, and depth in the case of threaded implants [10, 11]. In consideration of increasing surfaces appointed for osseous integration, threaded implants are generally preferred to smooth cylindrical ones [12].

The use of screw-type implants increases contact area and improves implant stability [13]. Other designs, such as the stepped implant and the tapered body of threaded implant, have also been proposed to mimic the root anatomy and to enhance the bony support in spongy bone, thereby creating a favorable load distribution [14, 15]. In addition, the thread size, thread profile, and surface roughness may affect the stress pattern in the surrounding bone [16–18].

Otherwise, occlusal loading may often be applied to an implant within 48 h after implant placement [19]. Nevertheless, the effectiveness of an immediately loaded implant is less predictable than that of the delay-loaded implant [20]. The main concern is the occurrence of fibrous encapsulation instead of osseointegration around implants [21].

The objective of this research is to compare the biomechanical effects of the immediately loaded dental implants and the surrounding bone tissue with various abutments (straight and angled), implant threads (trapezia tooth, rectangle tooth, and saw tooth), and healing methods (submerged and nonsubmerged) using a three-dimensional finite element analysis, accounting for the interaction between the dental implants and the supporting bone tissues. Three contact models and four types of loading conditions are used to simulate different integration qualities at the implant-bone interface during the osseointegration process. Extensive numerical simulation results show the influences of compositional profile, occlusal force orientations, and preload types on the static and dynamic behavior of the implant/bone system.

## 2. Materials and Methods

### 2.1. CAD Modeling

**Figure 1:**
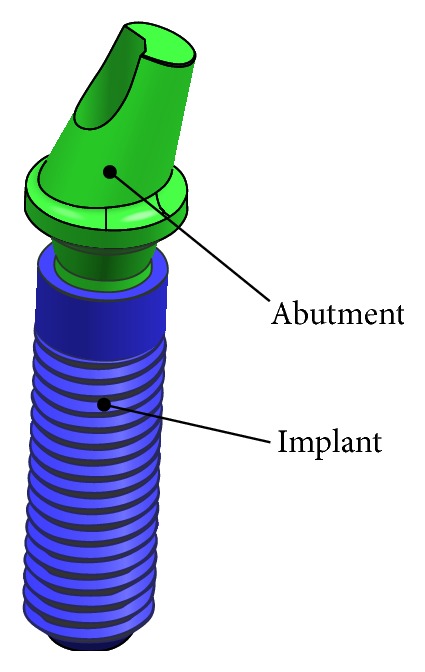
Dental implant system.

**Figure 2:**
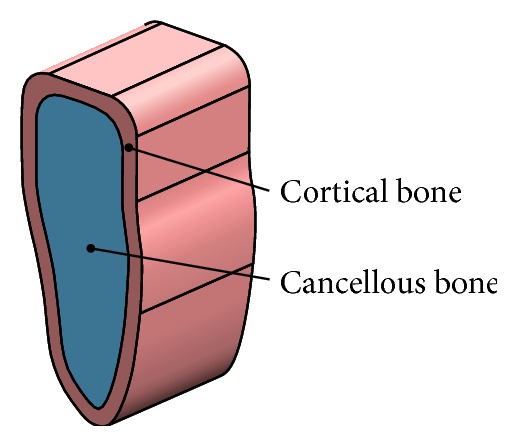
Surrounding bone tissues.

The three-dimensional geometrical model of the dental implant (Figure 1) and surrounding bone system (shown in Figure 2) was created using the CAD software Unigraphics NX 4.0 (Siemens PLM Software Inc., Germany). The geometry of the adult mandible took the shape created from CT database through image segmentation and spline reconstruction with STP format [24].

**Figure 3:**
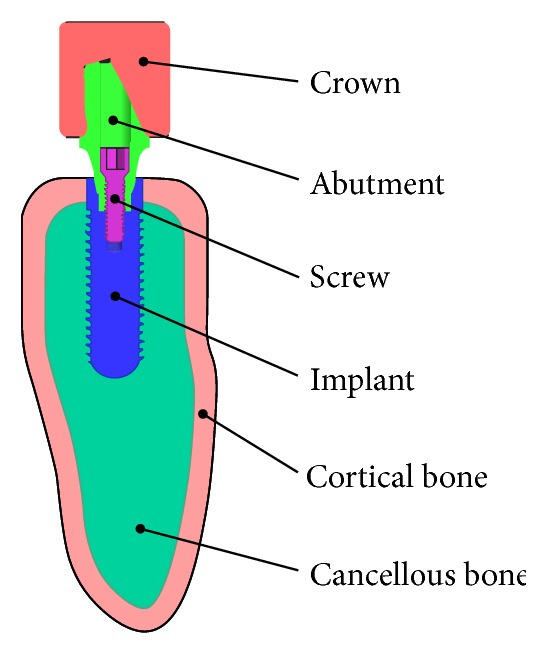
Dental implant/bone system.

The dental implant/supporting bone system comprised abutment, an implant, an internal screw connecting the abutment and implant, and prosthetic crown duplicated from the molar, surrounding cortical bone and cancellous bone in the mandibular section (Figure 3).

**Figure 4:**
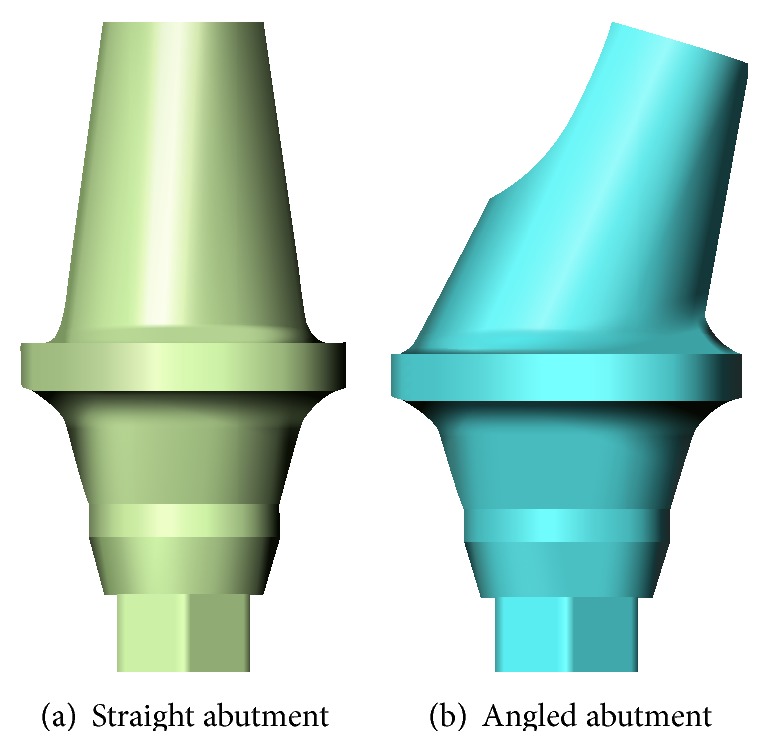
Abutment category.

As shown in Figure 4, the abutments were divided into straight abutment (shorted for “St”) and angled abutment (shorted for “An”), respectively. The maximum diameter was 5.1 mm, wearing gingiva length was 5 mm, and the inclined angle of angled abutment was 15° (Straumann Product Catalog 2012, Straumann AG, Switzerland).

In dentistry, platform switching was a method used to preserve alveolar bone levels around dental implants. A narrower abutment diameter for a given implant platform diameter was used [25].

**Figure 5:**
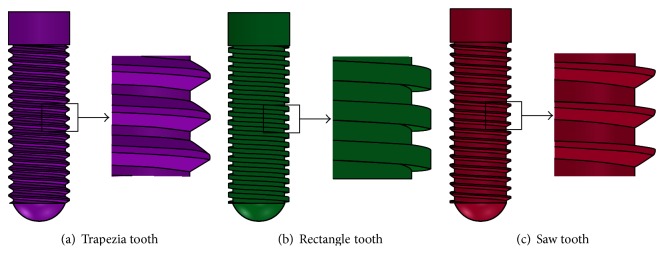
Thread category of the implant.

The diameter and length of the implant were 4.1 mm and 14 mm, respectively (Straumann Product Catalog 2012, Straumann AG, Switzerland).  Figure 5 illustrated external thread of the implant comprising trapezia tooth shorted for “Tr” (pitch P was 0.6 mm, thread depth was 0.5P, and thread angle was 30°), rectangle tooth shorted for “Re” (pitch P was 0.6 mm, thread depth is 0.5P, and thread angle was 0°), and saw tooth shorted for “Sa” (pitch P is 0.6 mm, thread depth was 0.75P, face flank angle was 3°, and nonface flank angle was 30°).

**Figure 6:**
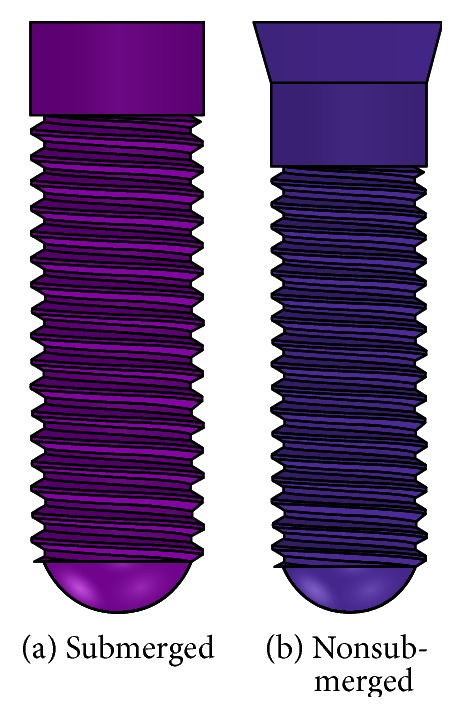
Healing method.

In the connection of the implant and the abutment, we adopted internal hexagon and Morse taper.  Figure 6 depicted two healing methods of submerged one shorted for “Su” (smooth neck height was 1.2 mm) and nonsubmerged one shorted for “Ns” (smooth neck height was 1.2 mm, the inclination angle was 15°, and total height was 2.0 mm) (Straumann Product Catalog 2012, Straumann AG, Switzerland).

**Figure 7:**
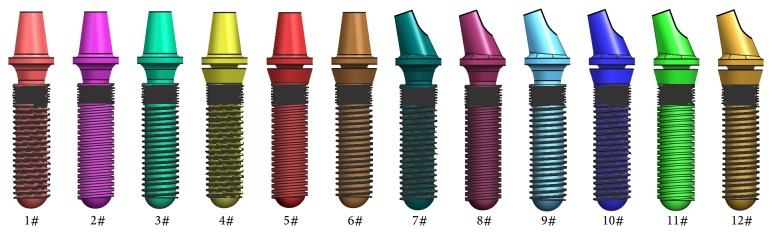
3D model of 12 dental implant systems.

According to the various structured abutments, implant threads, and healing methods, 12 combinations of the dental implant systems were exhibited (Figure 7 and Table 1).

### 2.2. Finite Element Modeling

**Figure 8:**
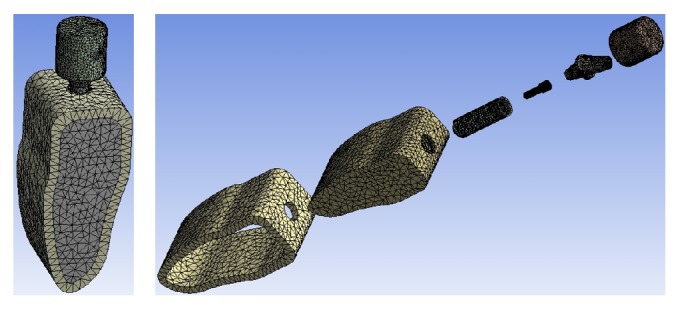
Finite element mesh view.

All 12 models described above were combined using Boolean operations, and the parasolid format of the solid model was then imported into ANSYS Workbench 14.0 (ANSYS, Inc., USA) to generate the FE model (Figure 8) using 10-node tetrahedral *h*-elements (ANSYS SOLID187 elements).

The convergence of the FEM analysis depended largely on the mesh grid. A standard convergence study was conducted by FEM analysis for mesh grids with different mesh refinement levels. A refined mesh was used in the threaded areas and the surrounding bone. For mesh grid, the relative errors for the maximum Von Mises stress in the implant system and the surrounding bone were computed as the percent differences between the current stress values and their counterparts predicted by the previous trial run. The calculation was considered to be convergent and the mesh grid was accepted when the relative errors were less than 1%. Number of total nodes is listed in Table 1, respectively.

### 2.3. Materials and Load Conditions

The abutment, implant, screw, cortical bone, and cancellous bone were treated as isotropic homogeneous linear elastic materials.  Table 2 listed Young's modulus (*E*), Poisson's ratio (*υ*), and Tensile Strength (Ts) of the materials used in the numerical examples. Because the elements were quite small, the material properties were assumed to be constant within each element.

**Figure 9:**
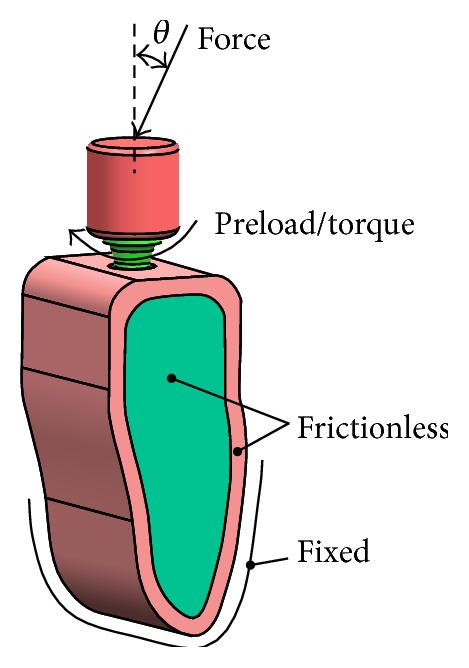
Load conditions of dental implant-bone tissue.

The bottom of the mandible was treated as fixed boundaries, and both side planes were frictionless, which was normal constraint (Figure 9). Two different contact models (“bonded” and “frictional”) are used to simulate different integration qualities at the implant and the supporting bone tissues during the osseointegration process. Using contact type of frictional to describe the integration quality among the abutment, implant, and screw interface and among implant, cortical bone, and cancellous bone interface (Table 3), the friction coefficient was 0.5 and 0.4, respectively [26]. Frictional contact implied that a gap between the implant and the peri-implant part might exist under an occlusal force. The rest of the contact surfaces were Bonded contact (Table 3). The “bonded” type simulated perfect osseointegration in which the implant and the surrounding parts were fully integrated so that neither sliding nor separation in the implant-bone interface was possible.

Based on oral physiology, four types of loading conditions (Figure 6) were simulated:

(1) A vertical occlusal force of 100 N (*θ* = 0) applied on the crown top surface [4], a preload of 200 N applied to the screw [27].
(2) A vertical occlusal force of 100 N (*θ* = 0) applied on the crown top surface [4], a torque of 0.2 N·m applied to the screw [27].
(3) An inclined occlusal force of 100 N (*θ* = 15°) applied on the crown top surface [4], a preload of 200 N applied to the screw [27].
(4) An inclined occlusal force of 100 N (*θ* = 15°) applied on the crown top surface [4], a torque of 0.2 N·m applied to the screw [27].

## 3. Results

Figure 7 gave the Von Mises stress distributions of the typical dental implants and the surrounding bone tissues under loading condition (1), (2), (3), or (4), respectively.

**Figure 10:**
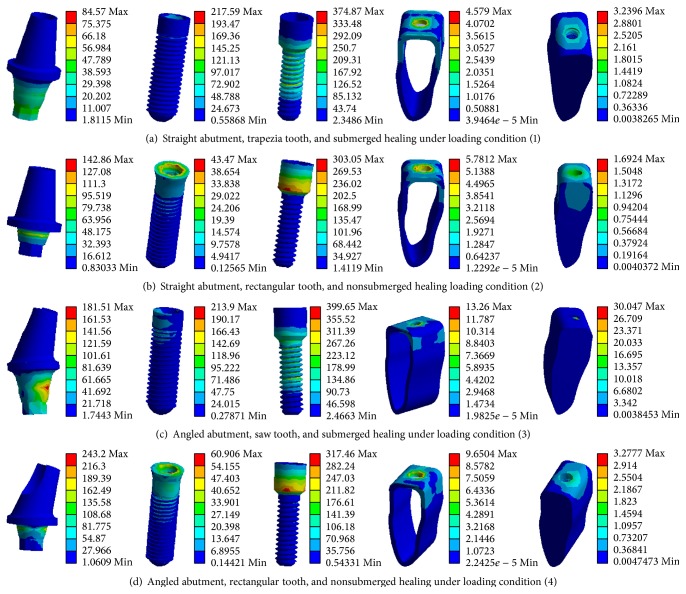
Stress distributions in the typical dental implants and the surrounding bone tissues.

As shown in Figure 10, the stress was mainly concentrated at the inner hexagon positioning junction because the force was just applied only on the contact surface. Application of the preload or torque applied to the screw resulted in the stress concentration on the screw, and fatigue failure would occur in the process of long-term use. The stresses in the cortical bone and cancellous bone, which were conjoint with implant, were relatively small due to the design concept of platform switching, which could reduce the stresses gradually at junction between the implant and the surrounding bone tissues, thus avoiding bone level being decreased in the long-term use.

**Figure 11:**
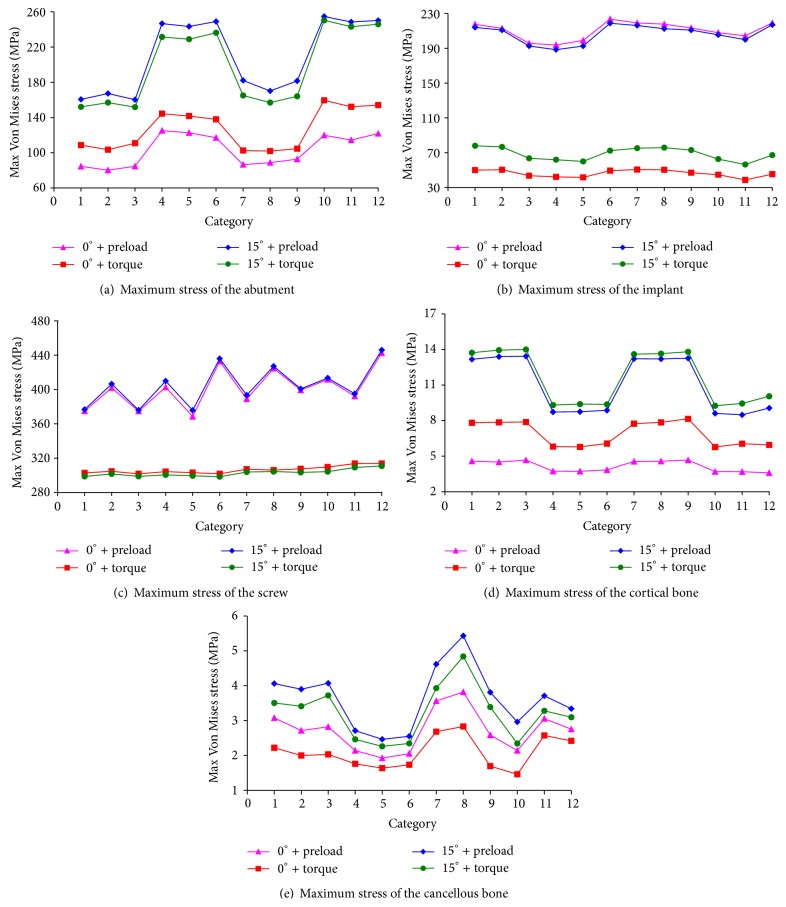
Maximum stress distributions of the dental implants and surrounding bone tissues.

Then we compared the maximum Von Mises stress distributions of 12 types of the dental implants and surrounding bone tissues (Figure 11).

Figure 11(a) exhibited the stress distribution of the abutment. When vertical force was applied on the crown, the abutment stress of the torque applied to the screw was larger than that of the preload condition while in the inclined force the abutment stress of the preload applied to the screw was larger than that of the torque condition. Both in the preload and in torque condition, the abutment stress of the inclined force was significantly higher than that in the vertical force of the crown. Taken together, the abutment maximum stresses of 1#, 2#, 3#, 7#, 8#, and 9# were rather small.

Figure 11(b) presented stress distribution of the implant. In the preloaded screw application, the stress difference was small in both the vertical force and the inclined force conditions. In the torque condition, the implant stress in the inclined force was larger than that in the vertical force. Both in the vertical and the inclined force, the preloaded application had great effect on the implant stress. Taken together, the implant maximum stresses of 3#, 4#, 5#, 10#, and 11# were rather small.

Figure 11(c) depicted the stress distribution of the screw. Whether in the preloaded or in torque application, the vertical and inclined force of the crown application had little effect on screw stress. However, under same loading conditions, the screw stress of the preloaded screw had greater effect than the torque one. Taken together, the screw maximum stresses of 1#, 3#, 5#, 7#, 9#, and 11# were rather small.

Figure 11(d) represented the stress distribution of the cortical bone. The cortical bone stress was relatively small. Whether in the preload or in torque application of the screw, the cortical bone stress of the inclined force was larger than the vertical force while in the vertical force the torqued screw application had greater effect on screw stress than the preloaded one. However, in the inclined force application, the torqued screw had smaller effect on screw stress than the preload condition. Taken together, the cortical bone maximum stresses of 4#, 5#, 6#, 10#, 11#, and 12# were rather small.

Figure 11(e) showed the stress distribution of the cancellous bone. The cancellous bone stress was relatively small. Whether in the preload or in torque application of the screw, the cancellous bone stress of the inclined force was larger than that of the vertical force. However, under same load conditions, the preloaded screw had greater effect on screw stress than the torque condition. Taken together, the cancellous bone maximum stresses of 4#, 5#, 6#, 9#, 10#, and 11# were rather small.

## 4. Discussion

Stress fields around endosteal implants and the supporting bone tissues were closely related to the type of loading and implant geometry [4]. In order to realistically simulate the stress state of the implant/bone system, four types of loading conditions (Figure 9) were studied.

Our results showed that the stress was mainly concentrated at the inner hexagon positioning junction because of the force just applied on the contact surface. The application of the preloaded or torqued screw resulted in stress concentration on the screw. However, the stresses in the cortical bone and cancellous bone which were conjoint with implant were relatively small.

Under same loading direction of the crown, the stress influence on the torqued screw was greater than that of the preload condition in the abutment and cortical bone while the stress influence of the preloaded screw was greater than that of the torqued condition for the implant, screw, and cancellous bone. The reason was mainly that the torque acted on the upper inner surface of the hexagonal hole of the screw while the preload was applied to the lower outer surface of the screw.

Meanwhile, under same loading mode of the screw, the stress distributions of the abutment, cortical bone, and cancellous bone in the inclined force on the crown were larger than those in the vertical force, up to 2 to 3 times. However, as for the implant and screw, the stress influence with different loading direction applied on the crown was not large. It was mainly due to the fact that the vertical force made stress distribution of the surrounding bone uniform through the cross section and the thread of implant. While the inclined force generated shear force and bending moment on the implant, thus the stress concentration at the implant's neck and bone contact area has taken place.

In addition, the abutment stress of nonsubmerged implant was larger than that of the submerged one under same load conditions. However, the implant, cortical bone, and cancellous bone stresses of nonsubmerged implant were smaller than those of submerged one indicating that if an overload condition occurred during chewing, the abutment of nonsubmerged system and the implant of submerged system would be susceptible to be broken, which could affect the long-term retention rate of the implant system. Therefore, doctors and patients need to take certain protective measures in use.

Table 4 listed stress distributions of 12 combinations of the dental implants and surrounding bone tissues (The symbol of “+” meant the unit with minimum stress of the implant-bone tissues). It was seen from Table 4 that 5# was the best option, which was the straight abutments, rectangular tooth, and nonsubmerged dental implant system. Meanwhile, 3#, 4#, and 11# were also provided with a certain application value.

## 5. Conclusion

Under same loading conditions, the thread had no significant effect on the implant stress but a greater impact on the cancellous bone stress. The stress distributions of the abutment, cortical bone, and cancellous bone in the inclined force of the crown were larger than that in the vertical force. The abutment stress of nonsubmerged healing implant system was larger than that of the submerged healing one. However, the implant, cortical bone, and cancellous bone stresses of nonsubmerged implant system were smaller than those of submerged one.

In conclusion, a dental implant system characterised by a straight abutment, rectangular tooth, and nonsubmerged healing method is the optimal design.

## Figures and Tables

**Table 1:** 12 combinations of the dental implant systems.

Category	Abutment	Implant	Healing	Nodes
1#	“St”	“Tr”	“Su”	124,128
2#	“St”	“Re”	“Su”	123,676
3#	“St”	“Sa”	“Su”	123,684
4#	“St”	“Tr”	“Ns”	123,060
5#	“St”	“Re”	“Ns”	123,294
6#	“St”	“Sa”	“Ns”	124,433
7#	“St”	“Tr”	“Su”	129,202
8#	“An”	“Re”	“Su”	128,994
9#	“An”	“Sa”	“Su”	129,578
10#	“An”	“Tr”	“Ns”	127,706
11#	“An”	“Re”	“Ns”	128,938
12#	“An”	“Sa”	“Ns”	128,721

St: straight; An: angled; Tr: trapezia; Re: rectangle; Sa: saw; Su: submerged; Ns: nonsubmerged.

**Table 2:** Material properties used in this study.

Material	Region	*E* (MPa)	*υ*	Ts (MPa)	Reference
Titanium	Implant, abutment, screw	102,000	0.35	485	[22]
Porcelain	Crown	68,900	0.28	835	[22]
Cortical bone	Mandible	13,000	0.30	133.9	[23]
Cancellous bone	Mandible	690	0.30	56	[23]

**Table 3:** Contact methods.

	Abutment	Screw	Implant	Cortical bone	Cancellous bone
Crown	Bonded	—	—	—	—
Abutment	—	Frictional	Frictional	—	—
Screw	—	—	Frictional	—	—
Implant	—	—	—	Frictional	Frictional
Cortical bone	—	—	—	—	Bonded

**Table 4:** Stress comparisons of 12 implants-bone tissues.

		Dental implant-bone system					
		Abutment	Implant	Screw	Cortical bone	Cancellous bone	Frequency
Implant combinations	1#	+		+			2
	2#	+					1
	3#	+	+	+			3
	4#		+		+	+	3
	5#		+	+	+	+	4
	6#				+	+	2
	7#	+		+			2
	8#	+					1
	9#	+		+			2
	10#				+	+	2
	11#		+	+	+		3
	12#				+	+	2

The symbol of “+” meant the unit with minimum stress of the implant-bone tissues.
